# Minimally invasive versus open transforaminal lumbar interbody fusion: a prospective, controlled observational study of short-term outcome

**DOI:** 10.1007/s10143-022-01845-w

**Published:** 2022-09-06

**Authors:** Sebastian Hartmann, Anna Lang, Sara Lener, Anto Abramovic, Lukas Grassner, Claudius Thomé

**Affiliations:** Department of Neurosurgery, Medical University of Innsbruck, Anichstrasse 35, A-6020 Innsbruck, Austria

**Keywords:** MIS-TLIF, MIS techniques, Spondylolisthesis

## Abstract

Instrumented stabilization with intersomatic fusion can be achieved by open (O-TLIF) or minimally invasive (MIS-TLIF) transforaminal surgical access. While less invasive techniques have been associated with reduced postoperative pain and disability, increased manipulation and insufficient decompression may contradict MIS techniques. In order to detect differences between both techniques in the short-term, a prospective, controlled study was conducted. Thirty-eight patients with isthmic or degenerative spondylolisthesis or degenerative disk disease were included in this prospective, controlled study (15 MIS-TLIF group vs. 23 O-TLIF group) after failed conservative treatment. Patients were examined preoperatively, on the first, third, and sixth postoperative day as well as after 2, 4, and 12 weeks postoperatively. Outcome parameters included blood loss, duration of surgery, pre- and postoperative pain (numeric rating scale [NRS], visual analog scale [VAS]), functionality (Timed Up and Go test [TUG]), disability (Oswestry Disability index [ODI]), and quality of life (EQ-5D). Intraoperative blood loss (IBL) as well as postoperative blood loss (PBL) was significantly higher in the O-TLIF group ([IBL O-TLIF 528 ml vs. MIS-TLIF 213 ml, *p* = 0.001], [PBL O-TLIF 322 ml vs. MIS-TLIF 30 ml, *p* = 0.004]). The O-TLIF cohort showed significantly less leg pain postoperatively compared to the MIS-TLIF group ([NRS leg 3rd postoperative day, *p* = 0.027], [VAS leg 12 weeks post-op, *p* = 0.02]). The MIS group showed a significantly better improvement in the overall ODI (40.8 ± 13 vs. 56.0 ± 16; *p* = 0.05). After 3 months in the short-term follow-up, the MIS procedure tends to have better results in terms of patient-reported quality of life. MIS-TLIF offers perioperative advantages but may carry the risk of increased nerve root manipulation with consecutive higher radicular pain, which may be related to the learning curve of the procedure.

## Introduction

The treatment of lumbar degenerative spinal disk disease with or without segmental instability is still a matter of debate [32]. Nevertheless, the rate of lumbar fusion surgery is rising dramatically compared to other musculoskeletal surgical procedures [6]. From 2004 to 2015, there has been a 62% increase in the USA regarding lumbar fusion procedures for degenerative conditions, with approximately a 32% increase in the population rate [30]. Although surgical treatment has shown advantages compared to conservative treatment in degenerative spondylolisthesis [13, 33], patient self-reported outcome data after spondylodesis for degenerative conditions shows unsatisfactory results in 30–40% of patients in randomized controlled trials [4, 13]. Moreover, complication rates of up to 20% in the often elderly population were reported in multisegmental procedures [5]. Complication rates appear to increase with age, blood loss, duration of surgery, and the number of levels treated.

With the introduction of minimally invasive spine surgery (MISS), better outcomes were expected by substantially reducing tissue damage as well as achieving the same treatment goals compared to a traditional open approach [23, 56]. Over time, many different MIS-TLIF techniques were described, which differ in the type of access or in the type of retractor system used [28, 53]. In general, MIS-TLIF is defined by some key features including the use of a nonexpandable or expandable tubular retractor, performing a paramedian or lateral incision, and the use of a microscope or endoscope for visualization [28]. With these special techniques, it is possible to approach the same anatomic landmarks as in open lumbar surgery, without using big skin incisions with massive muscle trauma [23, 53, 56], leading to more favorable results regarding intraoperative blood loss, need for blood transfusions, shorter hospitalization times, and less postoperative pain [21, 25, 43].

Nowadays, MISS is constantly increasing. Analyses of the few comparative studies suggest that MISS, in comparison to open surgery, does not show any advantages with regard to the most common clinical outcome instruments like ODI and VAS at follow-ups of 6 month and longer [21]. These results indicate that potential advantages of MISS are mainly found in the early postoperative phase. Novel techniques, however, are often associated with significant publication bias and it has been argued that MIS-TLIF requires more nerve root manipulation [7] and/or they lead to insufficient decompression. These parameters have hardly been studied prospectively so far, although they may be most relevant for elderly and comorbid patients. These patients would benefit significantly from decreased blood loss and earlier mobilization postoperatively [42], but would suffer from increased radicular problems. These differences might also have a considerable socio-economic impact, as recent investigations report the majority of cost savings due to a more rapid mobilization and discharge after MISS [1, 15, 30, 41].

## Methods

The COMOSA (comparing a minimally invasive to open instrumented spondylodesis approach) study was designed as a single-center prospective cohort study and was intended as a preliminary study on evaluation tools for a randomized, controlled study. This study was approved by the local ethics committee (ID: UN4624; session number 310/4.6) to ensure the standards of good clinical practice (GCP) and was performed in accordance with the ethical standards as laid down in the 1964 Declaration of Helsinki. All participants signed a written consent form. Afterwards, study participants were subdivided into a minimally invasive (MIS) and an open (O) non-randomized cohort, so that the group assignment, whether MIS or O, was done by the surgeon. Only surgeons, who are experienced in both techniques, performed the operations. The O-TLIF (open transforaminal lumbar interbody fusion) group was treated using a traditional open procedure [16, 17]. The MIS-TLIF (minimal invasive transforaminal lumbar interbody fusion) group was treated by performing the essential steps of MIS-TLIF including decompression, with central or bilateral decompression (using the over-the-top decompression technique), discectomy, and interbody graft insertion using an expandable retractor system (Pipeline; DePuy Synthes Spine, PA, USA), as well as pedicle screw and rod placement (VIPER® 2 MIS Spine System; DePuy Synthes Spine, PA, USA) [28]. The interbody cage and the corresponding screws were placed from one side through a mini-open approach using an oblique cage system (CONCORDE® interbody system; DePuy Synthes Spine, PA, USA). The contralateral screws were placed percutaneously. The postoperative management was not affected by the treatment group and followed institutional standards. The purpose of the study was the observation of the surgical and the early postoperative period up to 12 weeks. A set of outcome instruments that are well established and validated in geriatric patients such as the Timed Up and Go test (TUG) and the Barthel Index (BI) were chosen to investigate the postoperative short-term course [40, 48].

### Inclusion criteria

Inclusion criteria involved the participants’ age between 18 and 99, and clinical signs of lumbar degenerative disk disease from L1 to S1, confirmed with magnetic resonance imaging (MRI) and/or computed tomography scans (CT). Either central canal stenosis, lateral recess stenosis or foraminal stenosis which lead to radiculopathy, defined as pain and/or motor weakness or paralysis and/or paraesthesia in at least one specific nerve root distribution from L1 to S1, and/or neurogenic intermittent claudication, defined as pain and/or weakness and/or abnormal sensations in the legs during walking or prolonged standing, was diagnosed. Symptoms had to include at least one of the following criteria, like leg pain with a minimum of 30 mm on the 100 mm visual analog scale (VAS), decreased muscle strength of at least one level on the 0–5 Medical Research Council (MRC) scale, and abnormal sensation, including hypoesthesia, paresthesia, and hyperesthesia. Radiological and clinical findings had to indicate decompressive surgery and instrumented mono- or bi-segmental spondylodesis with a pedicle screw/rod-based system and an intervertebral cage (TLIF) according to the proposed literature [16, 17].

Further inclusion criteria were at least one of the following pathologies attributed to low back pain with a minimum of 40 mm on the 100 mm on the visual analog scale: degenerative disk disease Pfirrmann V, osteochondrosis Modic type I, spondylolisthesis Meyerding I° and II°, lumbar degenerative scoliosis Cobb < 10°, or expected iatrogenic destabilization. Moreover, the patients had to be unresponsive to non-operative treatment for a minimum of 3 months, including at least physiotherapy, pain medication, and local injections. Furthermore, the presence of progressive symptoms or signs of nerve root compression despite conservative treatment indicated fusion surgery. Exclusion criteria are listed in Table 1.

**Table 1:** Exclusion criteria

Exclusion criteria
Previous surgery (instrumented lumbar spinal surgery, cervical and/or thoracic spinal disease) to the extent that surgical consideration is likely or anticipated within 6 months after the lumbar surgical treatment
Other degenerative joint diseases (i.e., shoulder, hip knee) to the extent that surgical consideration is likely or anticipated within 6 months after or before the lumbar surgical treatment
Other physical diseases (e.g., neuromuscular disorders) before and/or within 6 months after lumbar surgical intervention which are able to restrict study procedures (i.e., wheelchair bound)
Neoplasia as the source of symptoms fixed or permanent neurological deficit, unrelated to the lumbar spine disease
Active or chronic infection, systemic or local (including HIV, AIDS, hepatitis)
Active malignancy defined as a history of any invasive malignancy, except non-melanoma skin cancer, unless the patient has been treated with curative intent and there have been no clinical signs or symptoms of the malignancy for a minimum of 5 years
Autoimmune disorder that impacts the musculoskeletal system (i.e., lupus, rheumatoid arthritis, ankylosing spondylitis)
Acute episode or major mental illness (psychosis, major affective disorder, or schizophrenia)
Physical symptoms without a diagnosable medical condition to account for the symptoms, which may indicate symptoms of psychological rather than physical origin
Recent or current history of substance abuse (drugs, alcohol, narcotics, recreational drugs)
Known allergy to titanium
3 or more vertebral levels requiring surgical treatment in the lumbar spine and clinically compromised vertebral bodies at the affected level due to current or past trauma (including osteoporotic fractures)

### Outcome parameters and course of the study

The overall follow-up period was 3 months, including a preoperative (baseline) visit, an intraoperative assessment, and six postoperative visits on the 1st, 3rd, and 6th day after surgery as well as 2, 4, and 12 weeks postoperatively. This tight follow-up schedule was chosen not to overlook temporary changes. The primary outcome measures included the VAS [3] for evaluating leg and back pain, and the TUG test [40] for functional gait assessment to detect spine-related disability. Secondary outcome parameters included spine-specific questionnaires incorporating the Oswestry Disability Index (ODI) [9], the Performance-based Barthel Index (PBI) [48], and the Core Outcome Measures index (COMI) [29]. For spine-related quality of life, an overall health status and the geriatric depression scale (GDS) were applied for patient’s self-assessment. Furthermore, the EuroQol-5D (EQ-5D) questionnaire was used [2]. The self-assessment questionnaire, containing ODI, COMI, GDS, health status, and EQ-5D, was filled out by patients preoperatively, on day 6 postoperatively as well as after 2, 4, and 12 weeks. Additionally, neurological status and back and leg pain according to the numeric rating scale (NRS) [8] were assessed. Regarding previous study results, the NRS might be the more reliable instrument to assess pain comparing VAS due to better compliance. Nevertheless, we evaluated both because of their association and ease of use [12, 19, 52]. For perioperative outcomes, intra- and postoperative blood loss (IBL and PBL) and intra- and postoperative complications were recorded.

### Statistical evaluation

Values are expressed by means ± standard deviation (SD). The Kolmogorov–Smirnov test was used for testing normal distribution. The unpaired Student *t* test and Mann–Whitney *U* test were performed to analyze differences in clinical and demographic characteristics and in clinical outcome variables. Frequencies were compared by the chi-square and Fisher’s exact tests. Spearman’s rho correlation (*r*) was determined to assess the relationship between clinical outcome and demographics. A *p* value < 0.05 was considered statistically significant. All statistical evaluations were performed with SPSS Version 21.0 (IBM Corp. Released 2012. IBM SPSS Statistics for Mac OS X, Version 21.0, NY: IBM Corp.). Figures were designed using Microsoft Excel (Version 15.36 for Mac OS X, Microsoft Corporation 2017, Redmond, USA).

## Results

### Demographic details

Thirty-eight patients treated with a lumbar fusion surgery were prospectively included in this trial. Twenty-three patients (60.5%) were treated with a one- or two-level open TLIF procedure (group O) and the remaining fifteen patients were treated with a minimally invasive TLIF procedure (group MIS). There were no significant differences in demographic patient characteristics (Table 2). The duration of surgery (DOS) tended to be shorter in the MIS group (179.0 ± 46.1 min) compared to the O group (197.9 ± 53.7 min; *p* = 0.428). Intraoperative loss of blood (ILB) was significantly lower in the MIS group (212.6 ± 178.1 ml) compared to the open counterpart (527.6 ± 315.1 ml; *p* = 0.001). The same was true for the postoperative loss of blood (PLB) through wound drain removed on the third postoperative day (30.0 ± 94.4 ml, MIS group; 321.5 ± 351.7 ml, O group [*p* = 0.004]). No major intraoperative complications in both groups occurred. Accidental durotomy occurred in both groups without statistically significant difference (O: 2/23, 8.7% vs. 1/15, 6.7%; *p* = 0.896).

**Table 2:** Demographic details of the patient cohort

		Group O, *n* = 23	Group MIS, *n* = 15	*p* value
Age	In years (SD)	59.1 (± 12.6)	53.7 (± 8.7)	n.s
Sex, *n* (%)	Male	5 (21.7)	4 (26.7)	n.s
	Female	18 (78.3)	11 (73.3)	
BMI	In kg/m^2^ (SD)	28.9 (4.9)	28.7 (4.8)	n.s
ASA score, *n* (%)	°1	4 (17.4)	3 (20.0)	n.s
	°2	13 (59.1.)	7 (46.7)	
	°3	4 (17.4)	5 (33.3)	
	°4	1 (4.3)	0 (0.0)	
Smoking, *n* (%)		9 (39.1)	9 (64.3)	n.s
Treatment indication, *n* (%)	Osteochondrosis	5 (21.7)	6 (40.0)	n.s
	Degenerative spondylolisthesis	12 (52.2)	3 (20.0)	
	Isthmic spondylolisthesis	6 (26.1)	6 (40.0)	
Treatment level, *n* (%)	L4/5	13 (56.5)	10 (66.7)	n.s
	L5/S1	7 (30.4)	5 (33.4)	
	Bi-segmental	3 (13.0)	0 (0.0)	
Duration of surgery	In minutes (SD)	197.9 (± 53.7)	179.0 (± 46.1)	n.s
Loss of blood	Intraoperative, ml (SD)	527.6 (± 315.1)	212.6 (± 178.1)	0.001
	Postoperative, ml (SD)	321.5 (± 351.7)	30.0 (± 94.9)	0.004
Intraoperative complications, *n* (%)	Accidental durotomy	2 (8.7)	1 (6.7)	n.s

*n* population, *SD* standard deviation, *Group O* open surgical procedure, *Group MIS* minimally invasive surgery, *ASA* American Society of Anesthesiologists Score, *BMI* body mass index, *n.s*. not statistically significant

### Performance-based values

**Fig. 1:**
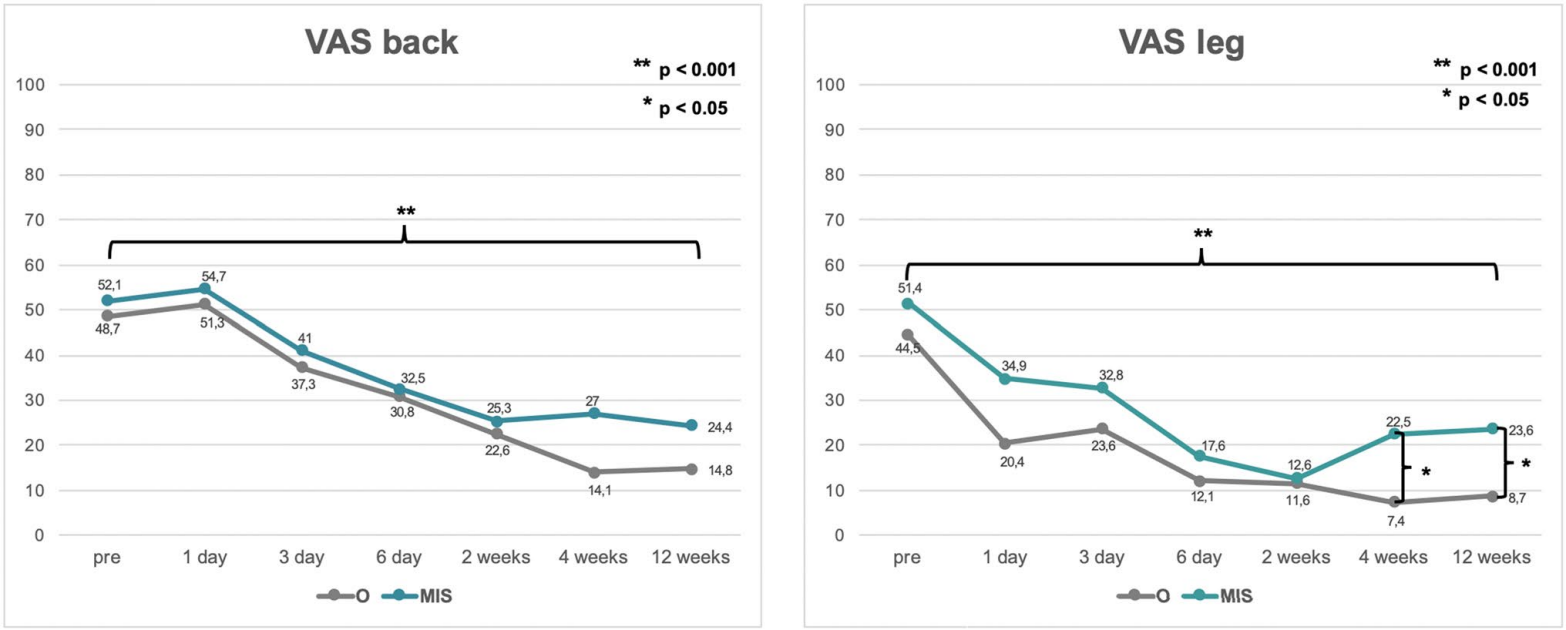
Valuation of pre- and postoperative VAS back and VAS leg. (O: open surgical procedure, MIS: minimally invasive surgery)

**Fig. 2:**
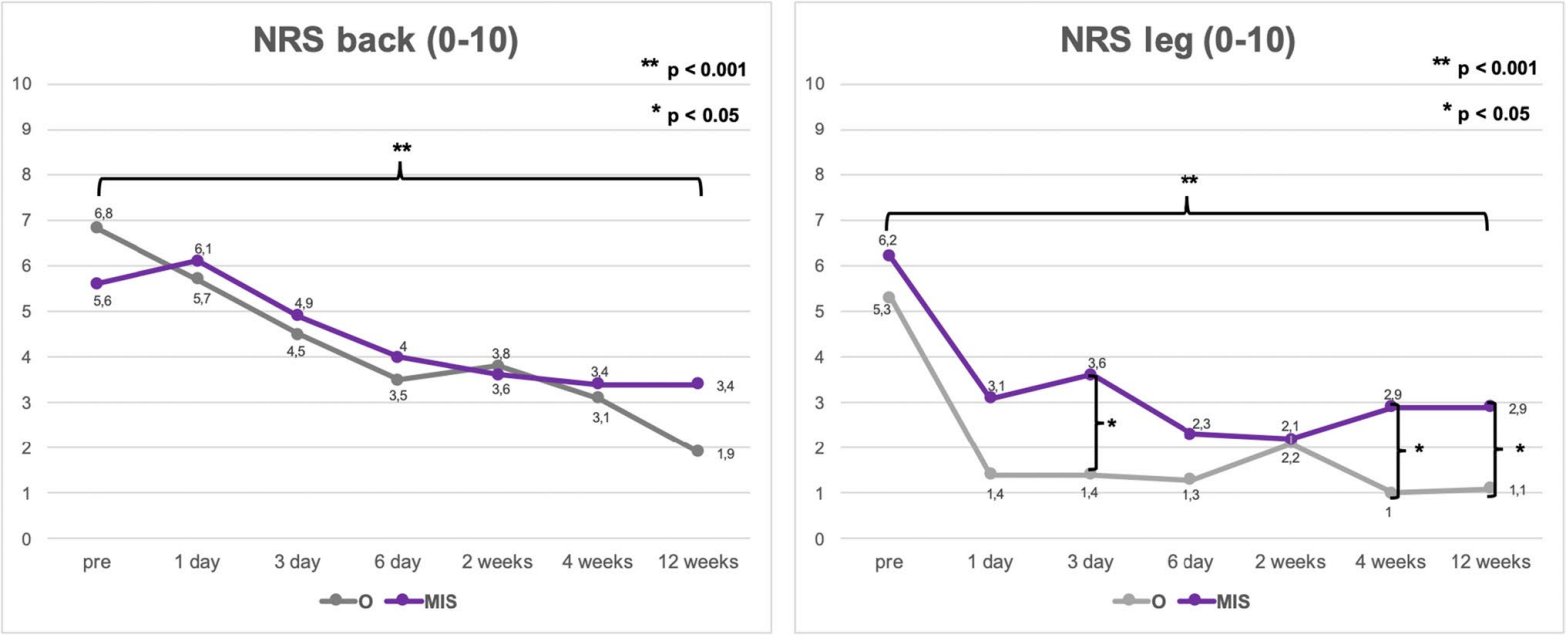
Valuation of pre- and postoperative NRS back and NRS leg. (O: open surgical procedure, MIS: minimally invasive surgery)

**Fig. 3:**
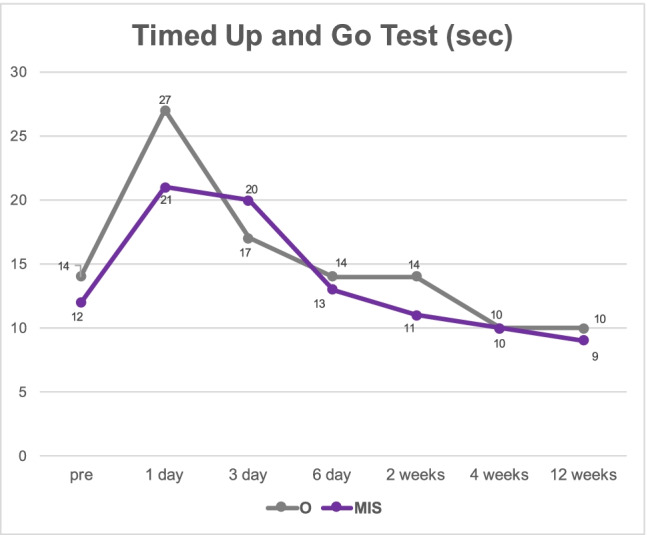
Valuation of pre- and postoperative Timed Up and Go (TUG) test for functional gait assessment. (O: open surgical procedure, MIS: minimally invasive surgery)

VAS leg and back pain, as well as NRS leg and back pain, improved significantly in both groups after 12 weeks (*p* < 0.01; Figs. 1 and 2). VAS and NRS leg pain of the side, where the cage was implanted, was rated significantly lower in the O group after 12 weeks postoperatively (O: 8.7 ± 17.2 vs. MIS: 23.6 ± 23.9 [*p* = 0.017]; O: 1.1 ± 2.2 vs. MIS: 2.9 ± 2.8 [*p* = 0.038], respectively). VAS and NRS back pain tended to improve faster in the MIS group without significant differences (*p* > 0.05; Figs. 1 and 2). Functionality, measured by the TUG, improved in both groups during the follow-up period. Group O tended to show higher disability on the first and third postoperative day (*p* > 0.05; Fig. 3).

### Patient self-reported outcome measurements

Analysis of the preoperative patient questionnaires demonstrated no statistically significant intergroup differences (Table 3). The overall ODI improved significantly in both groups between baseline and 12 weeks follow-up (O: pre: 47.6 ± 19, 12w: 22.0 ± 23; *p* = 0.017 vs. MIS: pre: 38.5 ± 12, 12w: 26.0 ± 13; *p* = 0.009). After 2 weeks postoperatively, the MIS group showed a significantly better improvement in the overall ODI (40.8 ± 13 vs. 56.0 ± 16; *p* = 0.05). All other time points postoperatively (6 days, 4 and 12 weeks) showed no significant differences. The EQ-5D-TTO and EQ-5D-VAS improved significantly from the preoperative visit to the visit 12 weeks postoperatively (O: *p* = 0.002/*p* = 0.003 vs. MIS: *p* = 0.010/*p* = 0.028). EQ-5D-VAS showed significantly better improvement in group O between the 6 days and 2 weeks follow-up (*p* = 0.017), whereas the EQ-5D-TTO showed higher levels of recovery in group O comparing week 4 and week 12 (*p* = 0.024). Both values adjusted over the time in both groups, still demonstrating better results in group O than in group MIS at the 12 weeks follow-up (TTO: O: 0.85 ± 0.2 vs. MIS: 0.68 ± 0.02; *p* = 0.007; VAS: O: 0.86 ± 0.2 vs. MIS: 0.76 ± 0.3; *p* = 0.043). The overall reported health status also improved significantly from the preoperative baseline to 12 weeks postoperatively showing higher values in the O group (79.1 ± 25; *p* = 0.001) than in group MIS (63.8 ± 28; *p* = 0.045). The GDS improved in both groups without significant differences but a tendency of improved results of the O group (*p* > 0.05). The COMI for leg and back pain showed significantly better results after 12 weeks postoperatively in group O (*p* = 0.001, for leg and back pain, respectively) as well as in group MIS (back: *p* = 0.010; leg: 0.018). It also tended to show faster improvement in group O when compared to group MIS, especially after 6 days, 2 weeks, and 12 weeks follow-up. The PBI showed statistically significant higher short-term improvement in group O than in group MIS, especially at the 6 days (*p* = 0.009) and 2 weeks follow-up (*p* = 0.0025), but showed no long-term improvements in neither group (*p* > 0.05).

**Table 3:** Overview of the patient-reported outcome measures (PROMs)

Pre				Pre - 6 days		6 days			6 d - 2 w		2 weeks			2 w - 4 w		4 weeks			4 w - 12 w		12 weeks			Pre - 12 w	
	O	MIS	*p*-value	O	MIS	O	MIS	*p*-value	O	MIS	O	MIS	*p*-value	O	MIS	O	MIS	*p*-value	O	MIS	O	MIS	*p*-value	O	MIS
				Δ p	Δ p				Δ p	Δ p				Δ p	Δ p				Δ p	Δ p				Δ p	Δ p
ODI sum	47.6 ± 19	38.5 ± 12	*n.s.*	*n.s.*	*n.s.*	49.5 ± 18	44.3 ± 13	*n.s.*	*n.s.*	*n.s.*	56.0 ± 16	40.8 ± 13	***0.050***	*n.s.*	***0.039***	39.8 ± 22	33.6 ± 13	*n.s.*	*n.s.*	*n.s.*	22.0 ± 23	26.0 ± 13	*n.s.*	***0.017***	***0.009***
EQ-5D-TTO	0.58 ± 0.3	0.51 ± 0.3	*n.s.*	*n.s.*	*n.s.*	0.55 ± 0.3	0.47 ± 0.2	*n.s.*	n.s.	n.s.	0.67 ± 0.3	0.54 ± 0.2	*n.s.*	*n.s.*	*n.s.*	0.75 ± 0.2	0.61 ± 0.2	***0.025***	***0.024***	*n.s.*	0.85 ± 0.2	0.68 ± 0.2	***0.007***	***0.002***	***0.010***
EQ-5D-VAS	0.61 ± 0.4	0.58 ± 0.3	*n.s.*	*n.s.*	*n.s.*	0.62 ± 0.3	0.61 ± 0.3	*n.s.*	***0.017***	*n.s.*	0.76 ± 0.2	0.66 ± 0.3	*n.s.*	*n.s.*	*n.s.*	0.84 ± 0.2	0.73 ± 0.2	*n.s.*	*n.s.*	*n.s.*	0.86 ± 0.2	0.76 ± 0.3	***0.043***	***0.003***	***0.028***
Health status	43.0 ± 23	49.2 ± 19	*n.s.*	*n.s.*	*n.s.*	51.1 ± 22	52.8 ± 22	*n.s.*	***0.030***	*n.s.*	64.7 ± 24	54.8 ± 22	*n.s.*	*n.s.*	*n.s.*	70.0 ± 27	66.0 ± 26	*n.s.*	*n.s.*	*n.s.*	79.1 ± 25	63.8 ± 28	*n.s.*	***0.001***	***0.045***
GDS	3.7 ± 4	4.6 ± 3	*n.s.*	*n.s.*	*n.s.*	3.0 ± 2.9	4.4 ± 2.7	***0.036***	*n.s.*	*n.s.*	2.9 ± 3.6	3.4 ± 2.6	*n.s.*	*n.s.*	*n.s.*	2.3 ± 3	3.7 ± 2	***0.024***	*n.s.*	*n.s.*	2.3 ± 3	2.9 ± 3	*n.s.*	*n.s.*	*n.s.*
COMI back	6.2 ± 2	5.5 ± 3	*n.s.*	*n.s.*	*n.s.*	5.2 ± 2	5.6 ± 2	*n.s.*	***0.002***	***0.006***	3.6 ± 2	3.9 ± 2	*n.s.*	***0.001***	*n.s.*	2.4 ± 2	4.1 ± 2	***0.013***	*n.s.*	***0.035***	2.4 ± 2	3.2 ± 2	*n.s.*	***0.001***	***0.010***
COMI leg	6.3 ± 3	5.3 ± 2	*n.s.*	***0.001***	***0.007***	4.3 ± 3	3.8 ± 2	*n.s.*	***0.008***	*n.s.*	2.5 ± 3	2.5 ± 2	*n.s.*	*n.s.*	*n.s.*	1.9 ± 2.3	3.4 ± 3	*n.s.*	*n.s.*	*n.s.*	1.7 ± 2	3.2 ± 2	***0.030***	***0.001***	***0.018***
PBI	98.3 ± 4	98.0 ± 5	*n.s.*	***0.009***	*n.s.*	90.0 ± 13	93.9 ± 8	*n.s.*	***0.025***	*n.s.*	95.8 ± 6	95.4 ± 6	*n.s.*	***0.029***	*n.s.*	98.9 ± 3	97.1 ± 6	*n.s.*	*n.s.*	*n.s.*	98.8 ± 3	98.9 ± 3	*n.s.*	*n.s.*	*n.s.*

*ODI sum* Oswestry Disability Index summary, *EQ-5D-TTO* time trade-of valuation, *EQ-5D-VAS* visual analog scale valuation, *GDS* geriatric depression scale, *COMI-back*, *-leg* Core Outcome Measures Index for the back and leg, *PBI* Performance-based Barthel Index, *n.s*. not statistically significant, *d* day, *w* week

## Discussion

Advantages and disadvantages of MIS-TLIF have been controversially discussed, as the results vary widely in the literature and publication bias may be an potential issue [14]. Before minimally invasive techniques in spinal surgery gained popularity, the traditional open approach represented the “gold standard” for lumbar instrumented fusion. This open approach involves large incisions and deattachment of healthy muscle tissue from bone resulting in increased muscle trauma, blood loss, and associated postoperative pain [39]. This muscle trauma leads to muscle edema, decreased muscular performance, and potentially denervation [24, 31]. These findings might be associated with prolonged hospital stays and increased postoperative complications [36, 46]. To overcome these problems, the minimally invasive approach for lumbar instrumented fusion has become increasingly attractive, as it provides potential benefits in terms of reduced paraspinal muscle trauma, resulting in decreased loss of blood, faster recovery rates, and reduced surgical site infection rates [39, 45, 55]. In contrast, the long-term observations comparing minimally invasive to open TLIF procedures failed to reveal significant differences, so that the beneficial effect of MIS procedures seems to be in the first weeks after surgery. Some studies examined functionality and pain after 3 months [35, 43], but the short-term period with analysis of multiple time points during the first 12 weeks postoperatively is still underreported. One study reported the average time until walking or standing-up postoperatively with MISS was 3.2 days compared to 5.4 days with open surgery [47]. Another study reported a significant reduction of muscle injury and systemic inflammatory markers during the acute postoperative period with MISS [25]. The authors suggested that MISS may play an important role in preventing medical morbidity after spinal surgery [25]. Others reported decreased pain, stress, fatigue, and mood disturbance 6 weeks postoperatively [50] and reduced surgical site infections [5, 37, 49] compared to patients who underwent open surgery. Postoperative narcotic use and return to work were found to be the most clinically relevant factors as both reduced twofold by MISS [1]. Despite these few reports about the postoperative short follow-up, there is no comparative study that focusses on the first 12 weeks postoperatively in detail.

Our study reports advantages of the MIS-TLIF procedure regarding operative time, intraoperative as well as postoperative blood loss, and some functional outcome scores. In terms of VAS and NRS, the MIS group revealed significantly more leg pain but a trend towards faster improvement of back pain compared to the open procedure. The patient-reported outcome measures (PROMs) demonstrated no statistically significant intergroup difference after 12 weeks.

Significantly more leg pain (VAS and NRS leg) of patients treated with MIS-TLIF was apparent at nearly all postoperative time points within the evaluated period of 12 weeks. The cause of increased leg pain in the MIS cohort may be explained by the greater nerve root retraction due to the minimally invasive approach. In the case of an open TLIF procedure, the facet joint is commonly widely resected and Kambin`s triangle may be approached with less retraction of the traversing and exiting nerve root as well as the dural sac without a predefined lateromedial angulation of a minimally invasive installed retractor [53]. By choosing a too medial MIS-TLIF approach, the dural sac and the traversing nerve root might be stressed. In case of a far lateral approach, Kambin’s triangle is approached in a flat angle leading to a small working corridor for cage implantation as well as potential irritation of the exiting nerve root. Two previous studies examined VAS leg after 6 and 24 months without significant differences [1, 38]. In contrast, immediate postoperative low back pain showed no significant differences between the two cohorts, even though back pain tended to improve faster in the minimally invasive treatment group potentially related to less muscle trauma. Our results are in line with the existing literature observing the long-term follow-up, as most studies have observed less postoperative pain in the MIS-TLIF cohort [10, 39] than in the open group mostly, without statistically differences.

In addition to that, the learning curve of MIS procedure cannot be neglected. MIS-TLIF requires mastering new techniques, which is thought to increase the length of operation due to the learning curve and also effects other perioperative factors such as complication rate [26, 34].

Functionality and disability are important factors after spinal surgery, so that most of the published studies evaluated the Oswestry Disability Index between MIS and open procedures between 3 and 24 months postoperatively [9]. To summarize their long-term findings (3 and 24 months postoperatively), MIS patients showed lower postoperative ODI scores compared to open-treated patients at nearly all study time points [11, 35, 47]. We can mostly agree with these findings, but a 1:1 comparison could not be drawn due to the lack of studies evaluating the short postoperative course. Focusing on the fact that the ODI depends on patients’ responsiveness, we additionally used the TUG test. There are recent studies demonstrating the reliability and the ease use of the TUG test [18, 51] for the measurement of functionality in case of spinal disorders. Considering the TUG, the MIS cohort also tended to outperform the open cohort. These findings might be also explained by the minimized surgical muscle trauma leading to reduced postoperative low back pain and consequently to a higher functionality state [39].

Summarizing the majority of studies in terms of surgical time, there were no significant differences between MIS and open TLIF [15, 26, 54], which coincide with our observation. Nevertheless, duration of surgery might only have a limited significance on the superiority of one technique, as it strongly depends on the surgeon’s routine [34].

Regarding peri- and postoperative blood loss as well as complication rate, we can support the results of prior studies [10, 22, 26], as in our observation the MIS cohort had a significantly reduced blood loss and no statistically significant difference in terms of complication rate. However, it should be mentioned that there are also many cofactors (multi-level fusion, preoperative hemoglobin, male gender, and body mass index) related to a higher loss of blood [20].

In terms of PROMs, they are less frequently recorded in the existing literature regarding the comparison of MIS and open TLIF procedures. In general, the most common PROMs consist of ODI, COMI, GDS, and health status questionnaire as well as the EQ-5D. Recent studies only reported on the Short-Form 36 (SF-36) [27, 44, 46], the Short-Form12 (SF-12) [27], and the EQ-5D [1, 37]. These findings showed no statistical significance in any of the reviewed studies in the long-term follow-up [15]. This observation also applied for the short-term follow-up within 12 weeks, without significant differences between both groups.

### Limitations

This prospective cohort study has several factors that may limit the relevance of the results. The lack of randomization of patients entails a possible selection bias. The low number of patients allows only a partial valid analysis of complication rates and differences between the two groups; additionally, there is no possibility for subgroup analysis. Documenting nerve root manipulation with leg pain as an indirect parameter carries the risk of subjective bias; nevertheless, intraoperative recording of manipulation is equally subjective. In view of strengths, there is no other prospective cohort study that conducted such early regular postoperative visits during the first 12 postoperative weeks and thus highlights the differences in this vulnerable phase especially in elderly and patients with comorbidities.

## Conclusion

This study showed similar postoperative outcomes for the open versus the MIS-TLIF approach, yet some relevant differences were demonstrated. Especially regarding functionality, the MIS cohort had better outcomes in ODI and TUG scores but seems to carry a risk of increased nerve root manipulation, as shown by increased postoperative leg pain. In experts, this risk may be negligible, but for beginners, it might be reduced by the use of a distractable cage and by the increased use of training simulators to get more experienced in MIS techniques. Nevertheless, further randomized, controlled trials and a long-term follow-up are necessary to provide advantages and disadvantages of the long-term effects of these two techniques.

## Data Availability

Not applicable.
